# Development and validation of the Measure of Indigenous Racism Experiences (MIRE)

**DOI:** 10.1186/1475-9276-7-9

**Published:** 2008-04-22

**Authors:** Yin C Paradies, Joan Cunningham

**Affiliations:** 1 Menzies School of Health Research, Institute of Advanced Studies, Charles Darwin University, Darwin, Australia; 2 Centre for Health and Society, School of Population Health, University of Melbourne, Melbourne, Australia

## Abstract

**Background:**

In recent decades there has been increasing evidence of a relationship between self-reported racism and health. Although a plethora of instruments to measure racism have been developed, very few have been described conceptually or psychometrically Furthermore, this research field has been limited by a dearth of instruments that examine reactions/responses to racism and by a restricted focus on African American populations.

**Methods:**

In response to these limitations, the 31-item Measure of Indigenous Racism Experiences (MIRE) was developed to assess self-reported racism for Indigenous Australians. This paper describes the development of the MIRE together with an opportunistic examination of its content, construct and convergent validity in a population health study involving 312 Indigenous Australians.

**Results:**

Focus group research supported the content validity of the MIRE, and inter-item/scale correlations suggested good construct validity. A good fit with *a priori *conceptual dimensions was demonstrated in factor analysis, and convergence with a separate item on discrimination was satisfactory.

**Conclusion:**

The MIRE has considerable utility as an instrument that can assess multiple facets of racism together with responses/reactions to racism among indigenous populations and, potentially, among other ethnic/racial groups.

## Background

In recent decades there has been emerging interest in the relationship between 'self-reported racism' and health. Self-reported racism (also referred to as 'perceived racism') is racism that is experienced or perceived and then reported by respondents in survey or interview settings [1]. A recent review found strong support for a relationship between racism and a range of poor health outcomes, especially mental ill-health [2]. Across the several hundred studies that have been conducted in this field of research to date, a number of instruments have been developed to measure racism for various ethnoracial groups (the terms 'ethnorace' and 'ethnoracial' are used in this paper to refer to race and/or ethnicity) [3]. However, of these instruments, only a select few have had their conceptual underpinnings described or been subject to validation beyond a check of internal consistency [1,4-12] Furthermore, studies of racism and health have been limited, to date, by: (1) a focus on African Americans to the exclusion of indigenous peoples, who also suffer from pernicious exposure to racism; and (2) a dearth of instruments which examine reactions/responses to racism [2,13]

The handful of other instruments that have been developed for use with Indigenous people suffer from a range of limitations including assessment using only a single-item [14], conflating exposure and affects [15] and not having a comprehensive and mutually exclusive assessment of racism [16-18]. With the exception of one instrument for which a factor analysis was performed and briefly reported [12], no other instrument used with Indigenous populations has undergone validation. In response to these identified lacunae as well as the need for an instrument which assesses multiple facets of racism [19,20], the Measure of Indigenous Racism Experiences (MIRE) was developed. The MIRE is a 31-item questionnaire designed with reference to a number of existing instruments [see Additional file 1] to assess self-reported racism across a range of dimensions for Indigenous people (the term Indigenous is used in this paper to refer to Australian Aboriginal and/or Torres Strait Islander people and is capitalized when denoting this specific social group rather than indigenous peoples more generally). Question 1 of the MIRE assessing inter-personal racism across nine mutually exclusive settings as well as, separately, from other Indigenous people. Question 2 consists of 11 items which assesses cognitive, affective and behavioural reactions to inter-personal racism while Question 3 includes a 4-item internalized racism scale and a 3-item systemic racism scale. Question 4 assesses respondents' race-consciousness while Questions 5 and 6 measure the salience of a respondents' ethnoracial identity within their social group and among strangers, respectively.

To our knowledge, the MIRE is the first such instrument to be developed for an indigenous population that assesses racism together with reactions/responses to such racism. This paper describes the development of the MIRE along with an opportunistic examination of its content, construct and convergent validity in a population health study.

## Methods

### MIRE development

Initial development of the MIRE was based upon a review of existing measures of self-reported racism, an exploratory focus group, conceptual dimensions of racism [21] and the personal experiences of the first author (an Indigenous Australian). The exploratory focus group assessed both the importance of racism as a form of oppression and the salient dimensions of racism experienced by Indigenous Australians. This focus group was moderated by the first author and involved four Indigenous professionals (one male and three females aged 25–50 years) living in Darwin, Australia. The resulting draft instrument was then reviewed by several experts in the field and by a range of Indigenous researchers for clarity, accuracy and completeness.

The content validity of the MIRE was then assessed in two confirmatory focus groups (moderated by the first author). As in the exploratory stage, focus group participants in this confirmatory research were Indigenous residents of Darwin, Australia, with one group consisting of eight working professionals (one male and seven females of middle-age) and another of five undergraduate students (two males and three females aged 20–30 years). No participants were involved in both the exploratory and confirmatory stages of this focus group research. Following an open-ended discussion of their experiences of racism (including reactions/responses), the MIRE was presented to participants for comment.

The final MIRE instrument consists of 31 items (presented as six multi-item scales) assessing exposure to inter-personal racism (Q1a-j), responses (Q2a-f) and reactions to racism (Q2g-k), internalized racism (Q3a-d), recognition of systemic racism (Q3f-g), race-consciousness (Q4), and salience of Indigeneity within social group (Q5) and among strangers (Q6). The items and scales of the final MIRE instrument along with response categories, conceptual underpinnings and sources for these items/scales are detailed in Additional file 1.

As shown in Additional file 1, the MIRE uses subjective (e.g. 'sometimes') rather than objective (e.g. 'once a week') frequency scales. Although some researchers have criticized this approach [22], subjective assessment is necessary to preclude differences in setting occupancy driving variation in the self-reported prevalence of racism across settings [23]. For example, a once a week experience of racism at work may be rated as occurring 'very often' if the respondent only works one day a week or 'sometimes' if the respondent works full-time. Furthermore, allowing respondents to determine the extent and impact of their own personal experiences of racism is particularly important for a phenomenon which is a fundamentally subjective experience [21].

Although the present tense used in this instrument suggests a focus on 'current' exposure, the MIRE does not include an explicit time frame for measuring exposure to racism. Explicit time frames on questionnaires are necessary in order to estimate the rate of exposure to racism and to avoid confounding time-series analysis [[24,25]:172–3]. Moreover, it has been suggested that lifetime or 'ever' questions are cognitively difficult and lead to underreporting [[25]:172–3]. However, no time period was specified in the MIRE because measuring "experiences of racism is best served by not restricting the period of time in which it occurred,"[11] given that the effects of traumatic experiences are potentially long lasting. A number of other instruments that measure racism do not specify an explicit time period, including the Perceptions of Racism Scale [5], the Telephone-Administered Perceived Racism Scale [10] and the Everyday Discrimination Scale [26]. As in the MIRE, these instruments seek to measure 'current' exposure as determined by respondents. This approach also avoids the problem of 'telescoping' in which respondents report experiences that occur outside the exposure period specified in a questionnaire [[24]:24].

### Administration of the MIRE in a population health study

The final MIRE instrument was administered during the first six months (between September 2003 and March 2004) of the Darwin Region Urban Indigenous Diabetes (DRUID) study. Eligible participants in the DRUID study were those aged 15 years and over who identified as Indigenous and who had lived (for at least six months) in a private dwelling within a defined geographic region in and around Darwin (a city of approximately 100, 000 people that is the capital of the Northern Territory (NT) of Australia) [see [27] for further details]. The study involved the collection of blood and urine samples, clinical and anthropometric measurements and the administration of questionnaires which assessed health status as well as socio-demographic, psychosocial and behavioural factors. It should be noted that the layout of the questionnaires used in the DRUID study resulted in MIRE Questions 4–6 being administered prior to and separate from MIRE Questions 1–3.

The psychometric, convergent and construct validity of the MIRE were examined using data from the 312 participants who responded to the MIRE as part of the DRUID questionnaires over the first six months of data collection. A majority of study participants were female (68%) and married (53%) while almost half (46%) owned or were purchasing their home and 10% had a university degree. Participants ranged in age from 15 to 81 years with a mean age of 41 years. The MIRE was interviewer-administered for 190 of these participants while the remaining 122 self-administered the questionnaire. All coding and analyses were conducted using Stata 8.0 for Windows (Stata Corporation, College Station, TX).

### Psychometric properties

With the exception of Q1 which is discussed below, the items in each MIRE scale were based on a single latent dimension and were measured in the same fashion. As such, Cronbach's α was used to assess the internal consistency of each MIRE scale [28]. With the exception of items Q2k and Q4-6, the underlying conceptual bases of MIRE items [see Additional file 1] were compared to empirical data from this study using factor analysis. As this study was exploratory in nature and utilized ordinal categories, exploratory principle components factor analysis was used to determine the empirical fit with underlying theoretical constructs. A principle components rather than principle axis approach was selected as it was assumed that the variance of MIRE items was potentially fully explicable by the factors derived during analysis [29]. The full five categories of Q1-3 were retained in factor analyses as component loadings and correlations may be under-estimated for variables with only two to three categories [30]. 'Not applicable' responses for Q1 were recoded as missing for these analyses. The Kaiser criterion was used to determine the number of components retained (i.e. components with eigenvalues equal to or greater than one were retained) [31]. Classical test theory was selected over item response theory as no substantial difference in performance has been found across these techniques and the former is characterized by a simpler underlying theoretical model and more robust assumptions [32,33].

### Construct validity

Inter-item and inter-scale associations were assessed using cross-tabulations and chi-squared tests. It was hypothesized that: (1) internalized and systemic racism measures would be inversely related; (2) increased reporting of inter-personal racism would be related to higher levels of systemic racism and lower levels of internalized racism; (3) higher levels of inter-personal racism and systemic racism as well as lower levels of internalized racism would each be associated with heightened race-consciousness (Q4); (4) reported experiences of racism from other Indigenous people would be associated with feeling less accepted by other Indigenous people (Q3a) and feeling less good about being an Indigenous person (Q3d); (5) more frequent use of adaptive responses to racism would be associated with higher levels of systemic racism and lower levels of internalized racism; (6) more frequent use of maladaptive responses to racism would be associated with higher levels of internalized racism and lower levels of systemic racism; and (7) there would be a concordance in responses to Q5 and Q6 (salience of Indigeneity within social group and among strangers, respectively).

Results that were significant at a p < 0.05 level (or marginally significant (0.05 <= p < 0.10) as indicated) are presented below when a consistent linear trend across the categories in question was evident (curvilinear trends were not considered) and if no cells had zero values and no more than 20% of cells had values less than five.

### Coding

For inter-item and scale analyses, items in MIRE Q1 and Q2 were recoded to a three-point scale (i.e. never/hardly ever, sometimes, often/very often), as were items in Q3 (i.e. strongly agree/agree, neither agree nor disagree, disagree/strongly disagree).

An average interpersonal racism score across the nine settings in Q1 was coded using the full five-point scale as follows: no inter-personal racism (all responses 'never'); low inter-personal racism (average response of 'hardly ever'); high inter-personal racism (average response of 'sometimes', 'often' or 'very often'). A composite variable consisting of the number of settings in which racism was experienced (0–9) was also coded and partitioned into quartiles.

After reverse coding Q3c, an average internalized racism score was coded from responses to Q3a-d as follows: no internalized racism (average response of 'strongly agree'); low internalized racism (average response of 'agree'); high internalized racism (average response of 'neither agree nor disagree' or 'disagree'). No participants had very high level of internalized racism (i.e. average response of 'strongly disagree').

An average systemic racism score was coded from responses to Q3e-g as follows: low systemic racism (average of 'strongly disagree'/'disagree'); moderate systemic racism (average of 'neither agree nor disagree'); high systemic racism (average of 'agree'); very high systemic racism (average of 'strongly agree').

Q4 was re-coded to a five-point scale ('never/once a year', 'once/month', 'once/week', 'once/day', 'hourly'/'constantly') and Q5 and Q6 were re-coded to a four-point scale ('no, hardly anybody/not many people', 'some people', 'yes, most people', 'yes, everyone'). 'Unsure' responses for Q5 and Q6 were recoded as missing.

### Convergent validity

An assessment of the MIRE's convergent validity was undertaken using an item from a separate stress checklist administered as part of the larger DRUID study questionnaire. This question was asked after MIRE Q4-6 and prior to MIRE Q1-3. Respondents were asked if, in the past 12 months, they or their family/friends had experienced discrimination (not specifically attributed to ethnorace) which affected them personally.

### Acceptability

The acceptability of the MIRE instrument to respondents was assessed in this study via an examination of missing responses to MIRE items and comparison against the missing response rates of other variables in the DRUID study.

## Results

### Acceptability

The exploratory focus group confirmed the importance of racism as a form of oppression for Indigenous Australians, while the two confirmatory focus groups supported the conceptualization, clarity and completeness of the MIRE (see [13] for details). No further modifications to the MIRE instrument were required as a result of these focus groups. The content validity of various MIRE items was also supported by the limited body of published research on the dimensions of racism experienced by Indigenous Australians [34-36].

Q4 (race consciousness) was the only question queried during confirmatory focus groups, with one participant expressing confusion as to its meaning. Feedback from interviewers and participants in the population health study indicated that this question continued to cause some confusion during administration of the MIRE. However, given that only ten participants had missing data for this item and that it displayed plausible patterns of association with other elements of the MIRE (see below), it does not appear that this confusion unduly affected the validity of this item. Nonetheless, until the reasons underlying such interpretational difficulty are identified, this item should be interpreted with caution.

### Psychometric Properties

The items in MIRE Q1a-i were designed to be mutually exclusive and comprehensive of the settings in which inter-personal racism may occur; as such, they are not based on one or more underlying theoretical constructs. The experience of inter-personal racism in any one of these settings does not necessarily increase the likelihood of experiencing inter-personal racism in any of the other settings. Therefore, although inter-dependence among these items is quite possible, internal consistency is not required for such a checklist and removal of items as a result of such consistency checking is not appropriate [23]. Nonetheless, for study participants with at least one non-missing item in Q1a-i (n = 301), these items showed a good level of internal consistency (α = 0.83), suggesting that participants who reported experiencing inter-personal racism in one setting tended to report inter-personal racism in other settings. Furthermore, the addition of Q1j (intra-racial racism) did not change the internal consistency of this question, suggesting that the experiences of inter-personal racism from Indigenous and non-Indigenous perpetrators were related.

Among the 212 participants who reported experiencing some inter-personal racism and who had non-missing data, Q2a-f (responses to inter-personal racism) had an alpha coefficient of 0.48, suggesting more than one underlying theoretical construct for these items. As shown in Table 1, factor analysis of these items produced two factors. As Q2a-f items loaded on both of these factors, an oblique rather than an orthogonal rotation was utilized. Factor one, which explained 30% of item variance, had a strong negative loading on Q2a and Q2f and a strong positive loading on Q2c-e.

**Table 1:** Principle component factor analysis of MIRE Q2a-f (responses to inter-personal racism)

Items	Factor 1	Factor 2	Item variance explained by factors one and two (1-uniqueness) (%)
alpha coefficient = 0.48			
Q2a	-0.36	0.46	37
Q2b	0.06	0.76	57
Q2c	0.39	0.71	59
Q2d	0.78	0.28	63
Q2e	0.79	-0.05	63
Q2f	-0.46	0.55	57
Total variance explained (%)	30	26	-

Both Q2a and Q2f assess passive maladaptive cognitive responses and hence would be expected to co-vary. Furthermore, the fact that Q2a and Q2f are non-adjacent items on the MIRE reduces the possibility that these findings are due to an order effect bias in which adjoining items are answered in a similar manner by respondents due to their proximity alone. The strong positive correlation of items Q2d and Q2e with factor suggests that this factor predominately captures outer-directed adaptive problem- and emotion-focused behavioural responses. As indicated by negative factor loadings for Q2a and Q2f, responses Q2d and Q2e are as theoretically different from Q2a and Q2f as possible, in that they are: (1) outer-directed (active) instead of passive; (2) adaptive instead of maladaptive; and (3) affective/behavioural instead of cognitive. Item Q2c assesses an inner-directed adaptive problem-focused behavioural response and, hence, is much more similar to Q2d and Q2e than to Q2a or Q2f.

Factor two, which explained 26% of item variance, displays the strongest positive loading on Q2b and Q2c as well as, to a lesser extent on Q2a and Q2f (see Table 1). As such, this factor predominately captures inner-directed adaptive problem-focused behavioural responses (assessed by both Q2b and Q2c). In contrast, the moderate positive loadings of factor two on the passive maladaptive cognitive responses (Q2a and Q2f) were unexpected. Although a small body of research suggests that trying to avoid racism (Q2b) or changing aspects of the self to prevent racism from occurring (Q2c) are adaptive [21], a recent study indicates these responses may be maladaptive among Indigenous people with respect to mental health [12]. If this is the case, it may explain the stronger than expected loading identified above.

For these same 212 participants, items Q2g-j (reactions to inter-personal racism) were characterized by a moderate internal consistency (α = 0.55). As shown in Table 2, a factor analysis of these items produced one factor which explained 45% of item variance and loaded highly on Q2g, Q2h and Q2j but poorly on Q2i. This factor explained less than 10% of the variance of Q2i, hence supporting the conceptual divergence of this item from items Q2g, Q2h and Q2j in that Q2i assesses an outer-directed empowered reaction to inter-personal racism whilst this factor captures disempowered reactions to racism (as assessed by Q2g, Q2h and Q2j).

**Table 2:** Principle component factor analysis of MIRE Q2g-j (reactions to inter-personal racism)

Items	Factor 1	Item variance explained by factor one (1-uniqueness) (%)
alpha coefficient = 0.55		
Q2g	0.75	56
Q2h	0.68	47
Q2i	0.31	9
Q2j	0.82	67
Total variance explained (%)	45	-

Responses from the 287 participants with non-missing items for Q3a-d (internalized racism) showed a very low degree of internal consistency (α = 0.23). As shown in Table 3, a factor analysis of these items produced two factors which explained 35% and 25% of the item variance, respectively. As these factors appeared to be orthogonal (i.e. no items appeared to load significantly on both factors), a varimax rotation was conducted. Results indicated that factor one primarily captured affective aspects of Indigenous identity (as assessed by Q3a and Q3d). As shown in Table 1, this factor also loaded to a reasonable degree on Q3b which assessed agreement with a statement of fact about the disadvantaged situation of Indigenous Australians. While factor one did not load to any significant degree on Q3c, this item was highly loaded on factor two with other items having weak loadings on this factor. Although there was no a priori reason to expect this, it appears that the factual item (Q3b) converges with the two affective items (Q3a, d) while the normative statement (Q3b) assesses a construct that was captured by factor two and was, hence, distinct from the other items in this subscale.

**Table 3:** Principle component factor analysis of MIRE Q3a-d (internalized racism)

Items	Factor 1	Factor 2	Item variance explained by factors one and two (1-uniqueness) (%)
alpha coefficient = 0.23			
Q3a	0.75	0.13	58
Q3b	0.51	-0.25	32
Q3c	0.03	0.96	93
Q3d	0.76	0.00	58
Total variance explained (%)	35	25	-

Responses from the 288 participants with non-missing items in Q3e-g (systemic racism) also evinced a moderate degree of internal consistency (α = 0.47). As shown in Table 4, a with a factor analysis of these items producing a single factor which explained 50% of item variance and loaded adequately on all three items in this scale, supporting the unidimensional nature of the systemic racism measure.

**Table 4:** Principle component factor analysis of MIRE Q3e-g (systemic racism)

Items	Factor 1	Item variance explained by factor one (1-uniqueness) (%)
alpha coefficient = 47		
Q3e	0.57	32
Q3f	0.84	71
Q3g	0.69	47
Total variance explained (%)	50	-

### Construct Validity

Hypothesis one (that internalized and systemic racism measures would be inversely related) was supported in that internalized and systemic racism were statistically significantly inversely associated, with participants who had high levels of internalized racism being less likely to have high levels of systemic racism. Only 7 of 284 participants had both high internalized racism and very high systemic racism scores. Hypothesis two (that increased reporting of inter-personal racism would be related to higher levels of systemic racism and lower levels of internalized racism) was partially supported in that a reduced likelihood of agreeing that Indigenous people should try to think and act more like other Australians (Q3c) was associated with more settings of reported racism (p = 0.06). However, neither systemic/internalized racism scores nor any other items in Q3 were statistically significantly associated with levels of inter-personal racism.

In support of hypothesis three (that higher levels of inter-personal racism and systemic racism as well as lower levels of internalized racism would each be associated with heightened race-consciousness (Q4)), as reported experiences of inter-personal racism increased, the frequency with which participants thought about being Indigenous (Q4) also increased. Agreeing that 'Indigenous people have less opportunities than other Australians' (Q3b) was also significantly associated with increased race-consciousness (Q4). However, neither systemic/internalized racism scores nor any other items in Q3 were statistically significantly associated with race-consciousness. Hypothesis four (that reported experiences of racism from other Indigenous people would be associated with feeling less accepted by other Indigenous people (Q3a) and feeling less good about being an Indigenous person (Q3d)) was wholly supported in that reported racism from other Indigenous people was associated with feeling less accepted by other Indigenous people (Q3a) as well as not feeling good about being an Indigenous person (Q3d).

There was strong support in this study for hypotheses five and six (that more frequent use of adaptive responses to racism would be associated with higher levels of systemic racism and lower levels of internalized racism and vice versa for maladaptive responses to racism). The maladaptive response of ignoring, forgetting about or accepting racism as a fact of life (Q2a) was associated with higher internalized racism (p = 0.07) and the adaptive response of talking about or expressing racism experiences (Q2e) was associated with lower internalized racism and higher reported systemic racism in general, as well as with agreeing that 'there is hardly ever anything good about Indigenous people in the media' (Q3g) (p = 0.07).

Agreeing that 'Indigenous people should try to think and act more like other Australians' (Q3c) was associated with more frequent attempts to change the way one is or the things that one does to prevent racism (Q2c). Given that Q3c is an item of the internalized racism scale, this finding supports the possibility considered above that Q2c may be maladaptive rather than adaptive among indigenous people. Similarly, those who reported the maladaptive response of keeping racism to themselves (Q2f) were more likely to have higher levels of internalized racism in general and to agree that 'Indigenous people should try to think and act more like other Australians' (Q3c). Being angry, annoyed or frustrated (Q2h) as an adaptive reaction to inter-personal racism was associated with lower internalized racism in general (p = 0.06) as well as with agreement that 'Indigenous people have less opportunities than other Australians' (Q3a) (p = 0.07) and that 'other Australians think they are better than Indigenous people' (Q3f).

Q5 provides information on how well-known a respondent's Indigeneity was within their social group, while Q6 attempts to capture the extent to which a respondent 'looks' Indigenous to strangers. Participants who 'look' Indigenous to strangers (Q6) should also 'look' Indigenous to those in their social group (Q5). In support of this conjecture (i.e. hypothesis seven: that there would be a concordance in responses to Q5 and Q6), a cross-tabulation of Q5 and Q6 shows that all participants who reported that most/all of the people they meet for the first time knew they were Indigenous (Q6) also reported that most/all of those in their social group know they are Indigenous (Q5).

### Convergent Validity

For those respondents with non-missing data for both the non-MIRE question on discrimination and the MIRE inter-personal racism score, there was a high degree of concordance between a positive response to the discrimination question and the inter-personal racism score. Only 6% of participants answered 'yes' to having been affected by discrimination while also reporting no inter-personal racism experiences. However, there was much less concordance between negative responses to these questions, with 64% of participants who reported some form of inter-personal racism in the MIRE also reported not being affected by discrimination. Put another way, only 25% of participants answered 'yes' to the discrimination question while about 70% of participants reported some experience of inter-personal racism across the nine settings included in the MIRE.

Reported discrimination and inter-personal racism evinced a similar pattern of association with other MIRE items/scales (given that the fewer 'yes' responses to the discrimination question reduced the statistical power to detect such associations). Participants who reported discrimination and/or inter-personal racism were more likely to report using adaptive behavioural responses (Q2d and Q2e) and to report experiencing all three disempowered affective (Q2g-i) and one somatic reaction to inter-personal racism (Q2k). Similarly, both discrimination and inter-personal racism were associated with race-consciousness (Q4). Furthermore, the discrimination item and MIRE Q1 items had similar patterns of association with health outcomes examined in the larger health study (see [13]).

Such findings suggest that discordance between the discrimination and racism items is unlikely to result from a variation in underlying constructs. In fact, terminology differences between these questions means that respondents who experienced racism that didn't affect them, their family or friends would appropriately report racism in the MIRE but not in the discrimination item. This could explain the main source of discordance between these measures. It is also possible that, as noted in other research [37,38], the use of ethnoracial terminology in the MIRE introductory text [see Additional file 1] lead to increased reporting of racism. Given that the proportion of participants reporting some level of racism in any one setting in MIRE Q1 (25–45%) is comparable to the 25% of participants who answered 'yes' to the discrimination question, it is also possible that a single item may not prompt respondents to report the full extent of their racism experiences. That is, increased reporting of racism in the MIRE may have resulted from prompting respondents' recall via explicitly listing a range of possible settings in which racism may have occurred.

The fact that the discrimination item assessed exposure over the past year while the MIRE included no explicit timeframe may also have contributed to the discrepancy in reported prevalence. The higher prevalence of racism reported in the MIRE could be explained if most experiences of racism had taken place more than a year prior to the administration of the MIRE. It is notable that the prevalence of reported discrimination was lower than the prevalence of inter-personal racism despite the fact that the former related to experiences that affected family/friends as well as that of the respondents themselves. Given these results, it is evident that further testing of the MIRE's convergent validity against other existing psychometrically-validated instruments is warranted.

### Acceptability

The proportion of missing responses for the 31 items in the MIRE ranged from 1% (Q5) to 10% (Q2g, h, j), with an average missing rate of 7% for the MIRE overall. In comparison, the missing response rate in the DRUID study was 15% for household income, 2% for marital status and 1% for self-assessed health status. This suggests that the MIRE had a moderate too high level of acceptability in relation to other variables measured in the DRUID study. As expected, across all MIRE items the rates of missing responses were 2–6 times higher for self- vs. interviewer administered questionnaires.

## Conclusion

Overall, the results from this study indicate that the MIRE has good content and psychometric validity. Construct and convergent validity were also reasonably well supported and the acceptability of MIRE items (as assessed via the missing data rate) appeared to be reasonable. In relation to construct validity, there was no evidence against the hypothesized relationships between MIRE constructs and it is possible that the small sample size in this study was responsible for the lack of statistical significance of some expected relationships.

Although the theoretical and methodological basis of the MIRE was supported in this paper, it should be noted that this research was not specifically designed to assess the validity of this instrument and hence the findings are opportunistic in nature. Given this, it will be important for future research to assess the reliability of the MIRE in relation to factors such as the method of collection, item order and ethnorace of interviewer as well as to examine test-retest reliability. As with other instruments in the field, further work could also examine if and how responses to the MIRE are affected by respondent characteristics such as neuroticism, hostility, cynicism, social desirability, impression management and self-deception/affirmation [see [39]].

Furthermore, although the validity of the internalized and systemic racism scales included in the MIRE were largely supported, it is unclear how accurately such manifestations of racism can be measured via individual self-reports. More focused and extensive research on internalized and systemic racism as experienced by indigenous peoples in Australia and elsewhere is required.

Indigenous people living in the Darwin area are broadly representative of other Indigenous people living in urban areas of Australia [27]. Compared with Indigenous people in the Darwin area, the participants in this study were more likely to be older and female, to have attained a university degree and to own or be purchasing (rather than renting) their home [34]. As such, it is recommended that further representative empirical research using the MIRE be conducted.

Having noted these limitations, this study has found that the MIRE is the first multiply-validated instrument designed to assess Indigenous peoples' experience of racism. Furthermore, this validation study has shown that each component of the MIRE has its own internal validity and could be used in isolation to measure interpersonal, internalized, or systemic racism as well as reactions and/or responses to racism, race-consciousness, and aspects of ethnoracial identity. As a whole, the MIRE could (with minor modifications) be utilized as an instrument that effectively assesses multiple facets of racism together with a diverse set of responses/reactions to racism among indigenous people and, potentially, among other ethnoracial groups.

## Competing interests

The author(s) declares that they have no competing interests.

## Authors' contributions

YP conceived of and designed the study, analyzed and interpreted the data and drafted the manuscript. JC participated in the design of the study, assisted in interpreting the data and helped to draft the manuscript. Both authors read and approved the final manuscript.

## Supplementary Material

Additional file 1 The Measure of Indigenous Racism Experiences (MIRE). Details of the question wording, response categories, conceptual bases and sources for the Measure of Indigenous Racism Experiences (MIRE). Click here for file
