# Supplementary material for: Development and validation of the Measure of Indigenous Racism Experiences (MIRE)
Source: Int J Equity Health. 2008 Apr 22;7:9. doi: 10.1186/1475-9276-7-9 (PMC2359753; doi:10.1186/1475-9276-7-9)
Supplement: Additional file 1 — The Measure of Indigenous Racism Experiences (MIRE). Details of the question wording, response categories, conceptual bases and sources for the Measure of Indigenous Racism Experiences (MIRE). [file 1475-9276-7-9-S1.doc]

## The Measure of Indigenous Racism Experiences (MIRE)

| **MIRE introductory text**  The following questions are about your experiences of being Aboriginal or Torres Strait Islander. In the questions the word ‘Indigenous’ is used to mean Aboriginal or Torres Strait Islander people including people who are both Aboriginal and Torres Strait Islander. Some questions ask about your experiences of ‘being treated unfairly’. People can be treated unfairly for all sorts of reasons. For these questions we only want to find out about times when you were treated unfairly because you are Aboriginal or Torres Strait Islander. Being ‘treated unfairly’ means being treated as if you were inferior, rudely, with disrespect, being ignored, insulted, harassed, stereotyped, discriminated against or having unfair assumptions made about you. |
| --- |

| **MIRE item** | **Response categories** | **Conceptual basis** | **Sources** |
| --- | --- | --- | --- |
| **Question 1** (Q1) How often are you treated unfairly because you are Indigenous in each of the following situations? (If the situation doesn't apply to you, please tell me) (a) At work or on the job?; (b) At home, by neighbours or at somebody else’s house?; (c) At school, university or other academic setting?; (d)While doing sporting, recreational or leisure activities?; (e) By the police, security personnel, lawyers or in a court of law?; (f) By doctors, nurses or other staff at hospitals or doctors’ surgeries?; (g) By staff of government agencies like Centrelink, ATSIC, etc.?; (h) By staff at restaurants, bars, shops, banks, motels, real estate agents, in taxis or when getting any other services?; (i) By other people on the street, at shopping centres, sporting events, concerts, nightclubs etc?;  (j) By other Indigenous people? | Never  Hardly ever  Sometimes  Often  Very often  This doesn’t apply to me | Assesses inter-personal racism across nine mutually exclusive settings as well as a non-mutually exclusive item on intra-racial racism from other Indigenous people | Q1a-i are based on [41] and the Schedule of Racist Events (SRE) [42] as well as the Telephone-administered Perceived Racism Scale (TPRS) [10] |
| **Question 2** (Q2) When you are treated unfairly because you are Indigenous how often do you: (a) Ignore it, forget about it or accept it as a fact of life?; (b) Try to avoid it in the future?; (c) Try to change the way you are or things that you do so that it won’t happen again?; (d) Try to do something about the people who did it or the situation in which it happened?; (e) Talk to other people like family or friends about it, or write, draw, sing or paint about it?; (f) Keep it to yourself?; (g) Feel ashamed, humiliated, anxious or fearful?; (h) Feel angry, annoyed or frustrated?; (i) Feel amused, contemptuous or sorry for the person who did it?; (j) Feel powerless, hopeless or depressed?; (k) Get a headache, an upset stomach, tensing of your muscles, or a pounding heart? | Never  Hardly ever  Sometimes  Often  Very often | Maladaptive passive cognitive responses: Q2a and Q2f. Adaptive inner-directed problem-focused behavioural responses: Q2b and Q2c. Adaptive outer-directed problem-focused behavioural response: Q2d. Adaptive outer-directed emotion-focused behavioural response: Q2e. Inner-directed disempowered active reaction: Q2g. Outer-directed disempowered reaction: Q2h. Outer-directed empowered reaction: Q2i. Inner-directed disempowered passive reaction: Q2j. Somatic reactions to racism: Q2k. Conceptual dimensions of reactions/responses are from [12] | Q2a and Q2d-f are based on [41] and Q2b-c on Q3 of the racism-encounter emotions and coping section of The Racism and Life Experiences Scales [43]. Reference to “write, draw, sing or paint” in Q2e is based on Q92H from the Koori young people’s health study [43, see also 44]. Q2g-j was informed by items in the TPRS [10], Q5 of the Reactions to Race Module in the US 2002 Behavioral Risk Factor Surveillance System (BRFSS) and the Perceived Racism Scale [46]. Q2k is taken from Q6 of the 2002 BRFSS |
| **Question 3** (Q3) Please indicate how much you agree or disagree with each of the following statements. There are no right or wrong answers. (a) I feel accepted by other Indigenous people; (b) Indigenous people have less opportunities than other Australians; (c) Indigenous people should try to think and act more like other Australians; (d) I feel good about being an Indigenous person; (e) Other Australians think that Indigenous people are better off because they get special treatment from the government; (f) Other Australians think they are better than Indigenous people; (g) There is hardly ever anything good about Indigenous people in the media (TV, radio, newspapers, etc.). Note: The phrase ‘less opportunities’ is used rather than the grammatically correct ‘fewer opportunities’ as the former was thought to be more easily understood by respondents. | Strongly agree  Agree  Neither agree nor disagree  Disagree  Strongly disagree | Q3a-d forms the internalized racism scale.  Q3a and Q3d assess a personal affective dimension, Q3b a factual dimension and Q3c a normative dimension of internalized racism.  Q3e-g forms the systemic racism scale which taps into common stereotypes and prejudices about Indigenous Australians | Q3a is based on Q108 from the Koori young people’s health study [43] while Q3b is based on Q11 of the Perception of Racism Scale [6]. Q3c and Q3e are based on Q16 and 20 of Section 4 in [47], Q3d is based on Q10 of the Racial Identity Attitude Scale [48:173] and Q3f is based on Q2 of Section four in [47]. Q3g is adapted from question 15 of the Index of Race-Related Stress [11] |
| **Question 4** (Q4) How often do you think about being  Indigenous? | Never  Once a year  Once a month  Once a week  Once a day  Once an hour  Constantly | Assesses respondents’ race-consciousness [49] | Q4 is based on [50] |
| **Question 5** (Q5) Do the people that you mix with know that you are Indigenous? | No, hardly anybody  No, not many people  Some people  Yes, most people  Yes, everyone  Unsure | Assesses the salience of a respondents’ ethnoracial identity within their social group | Q5 is adapted from Q73 of the Koori young people’s health study [44] |
| **Question 6** (Q6) Do people you meet for the first time know that you are Indigenous? | As for Question 5 | Assesses the salience of a respondents’ ethnoracial identity among strangers | Q6 is inspired by Q1 of the US 2002 BRFSS |
